# Diagnostic performance of the second-generation Wavelia microwave breast imaging system: a pilot clinical investigation

**DOI:** 10.1093/bjs/znaf242

**Published:** 2025-11-12

**Authors:** Eoin P Kerin, John P M O’Donnell, Sami M Abd Elwahab, Thomas O Butler, Luis Bouz Mkabaah, Angie Fasoula, Giannis Papatrechas, Petros Arvanitis, Luc Duchesne, Michael K Barry, Aoife J Lowery, Michael J Kerin

**Affiliations:** Department of Surgery, Lambe Institute for Translational Research, University of Galway, Galway, Ireland; Department of Surgery, Lambe Institute for Translational Research, University of Galway, Galway, Ireland; Department of Surgery, Lambe Institute for Translational Research, University of Galway, Galway, Ireland; Department of Surgery, Galway University Hospital, Galway, Ireland; Department of Surgery, Lambe Institute for Translational Research, University of Galway, Galway, Ireland; Department of Surgery, Lambe Institute for Translational Research, University of Galway, Galway, Ireland; Wavelia Healthcare, MVG Industries, Athens, Greece; Wavelia Healthcare, MVG Industries, Athens, Greece; Wavelia Healthcare, MVG Industries, Athens, Greece; Wavelia Healthcare, MVG Industries, Villejust, France; Department of Surgery, Galway University Hospital, Galway, Ireland; Department of Surgery, Lambe Institute for Translational Research, University of Galway, Galway, Ireland; Department of Surgery, Galway University Hospital, Galway, Ireland; Department of Surgery, Lambe Institute for Translational Research, University of Galway, Galway, Ireland; Department of Surgery, Galway University Hospital, Galway, Ireland

Mammographic screening programmes have significantly improved outcomes in breast cancer, reducing mortality rates by approximately one-fifth. Mammography is widely accepted as the gold standard for symptomatic disease detection in women over 40; however, shortcomings include poor sensitivity for invasive lobular carcinoma (ILC), low efficacy in younger and dense-breast cohorts, and compression-related discomfort which may impact screening uptake^1,2^. Although sonography is first line in younger patients and MRI offers high sensitivity in high-risk populations, both possess inherent limitations and logistical challenges for widespread implementation^2^.

Microwave breast imaging (MWBI) has emerged as a promising modality to complement conventional breast imaging techniques^2^. The Wavelia #2 MWBI System (Wavelia Healthcare, MVG Industries; Athens, Greece/Villejust, France) (Wavelia) is a second-generation, non-compressive, radiation-free device. The first-in-human feasibility study demonstrated sensitive lesion detection, high efficacy for ILC, and a favourable safety profile and user experience. These results informed refinements culminating in the novel Wavelia #2 prototype, accommodating a broader range of anatomical breast sizes^3^.

A single-arm prospective pilot study was conducted between February 2023 and May 2024 at University Hospital Galway’s Symptomatic Breast Unit to evaluate lesion detectability by Wavelia. At α = 0.05 and 80% power, 62 patients were required (Stage 1: *n* = 30; Stage 2: *n* = 32). Following recruitment, 62 women with a discrete breast abnormality underwent a Wavelia scan alongside routine standard-of-care work-up. Simon’s two-stage minimax design was employed to assess whether lesion detectability exceeded 75% (H₁). Ethical approval (NREC-MD-031) and HPRA authorization (CI52917) were obtained, and the trial was preregistered (NCT05757427). Full study design parameters, eligibility criteria and a schematic of the two-stage adaptive design are available in Supplementary Method*s* and *Fig. S1*.

Lesions were detected in 56/62 patients, yielding a sensitivity of 90% (95% c.i.: 80.5 to 95.5), exceeding the predefined performance threshold. Performance remained high across lesion subtype (that is benign or malignant). Notably, lesion detectability was highly accurate in young and pre-menopausal patients, and women with dense and extremely dense breasts. All lesions ≤15 mm were accurately detected by the system, and sensitivity was consistent irrespective of biopsy clip presence. Detailed subgroup results are shown in *Table 1*.

**Table 1:** Lesion detectability overall per-patient and per-lesion, by age group categories, by breast density category, and by presence of biopsy clip

Category	Benign (*N* = 30)	Malignant (*N* = 32)	Total (*N* = 62)
Overall (per patient)	29/30 (97%; 95% c.i.: 83.3–99.4)	27/32 (84%; 95% c.i.: 68.3–93.1)	56/62 (90%; 95% c.i.: 80.5–95.5)
Overall (per lesion)	33/37 (89%; 95% c.i.: 79.3–99.1)	27/33 (82%; 95% c.i.: 69.1–94.6)	60/70 (86%; 95% c.i.: 77.1–94.3)
**Age group**			
<40 years	15/15 (100%)	6/7 (86%)	21/22 (96%)
>40 years, pre-menopausal	11/11 (100%)	13/14 (93%)	24/25 (96%)
Post-menopausal	3/4 (75%)	8/11 (73%)	11/15 (73%)
**Breast density category**			
Fatty breasts (BI-RADS A or B)	4/5 (80%)	9/11 (82%)	13/16 (81%)
Dense breasts (BI-RADS C)	6/6 (100%)	9/10 (88%)	15/16 (94%)
Extremely dense breasts (BI-RADS D)	8/8 (100%)	9/11 (82%)	17/19 (90%)
No mammogram	11/11 (100%)	—	11/11 (100%)
Fatty breasts (BI-RADS A or B); ≤15 mm	1/1 (100%)	2/2 (100%)	3/3 (100%)
Dense/extremely dense breasts (BI-RADS C or D); ≤15 mm	6/6 (100%)	1/1 (100%)	7/7 (100%)
No mammogram; ≤15 mm	4/4 (100%)	—	4/4 (100%)
**Biopsy clip**			
Biopsy clip in situ	15/15 (100%)	26/30 (87%)	41/45 (91%)
No biopsy clip in situ	14/15 (93%)	1/2 (50%)	15/17 (88%)

*N*, number of patients. Values are *N* (%).

These results highlight Wavelia’s potential utility as an adjunct or alternative to contemporary breast imaging modalities. In the current paradigm, low mammographic sensitivity in dense-breast cohorts is confounded by this population’s breast cancer risk: four- to six-fold greater than those without dense breast tissue. Although ultrasound may improve accuracy in this cohort, this benefit is attenuated by a low positive predictive value^1^. Wavelia’s robust lesion detectability rates in these anatomically challenging and high-risk subgroups underscores its potential to enhance breast imaging in current practice.

Integration of AI will further strengthen diagnostic pathways, but a pragmatic approach is fundamental to improving patient outcomes in this regard^4^. Whereas competitor MWBI models produce limited-resolution images or bypass image reconstruction entirely for machine-learning models, Wavelia combines AI semi-automated lesion detection with radiologist expertise by generating highly interpretable true volumetric 3D images. This combination yielded sensitivity rates in this study exceeding those for counterpart models in early-phase studies, reported in the region of 70–80%^5^.

This pilot study was limited by its modest single-centre sample size, with 11 patients excluded following screening (*Table S1*). Additionally, a high proportion of lesions marked with biopsy clips prior to Wavelia imaging may confound detectability. Nevertheless, Wavelia accurately identified lesions across age, breast density, and lesion-type subgroups. High performance in younger women and those with dense breasts, cohorts by conventional imaging modalities, rationalizes the system’s potential to enhance screening and diagnostic pathways in future practice. Multicentre trials are warranted to validate performance across larger, more diverse cohorts.

## Supplementary Material

znaf242_Supplementary_Data

## Data Availability

The data underlying this article are not publicly available due to institutional regulations. Requests for access may be considered by the corresponding author, subject to institutional approvals.
