# Supplementary material for: Diagnostic performance of the second-generation Wavelia microwave breast imaging system: a pilot clinical investigation
Source: Br J Surg. 2025 Nov 12;112(11):znaf242. doi: 10.1093/bjs/znaf242 (PMC12605771; doi:10.1093/bjs/znaf242)
Supplement: znaf242_Supplementary_Data [file znaf242_supplementary_data.docx]

**Title**

Diagnostic Performance of the Second-Generation Wavelia Microwave Breast Imaging System: A Pilot Clinical Investigation

Authors

Eoin P. Kerin^1^, John P.M. O’Donnell^1^, Sami M. Abd Elwahab^1,2^, Thomas O. Butler^1^, Luis Bouz Mkabaah^1^, Angie Fasoula^3^, Giannis Papatrechas^3^, Petros Arvanitis^3^, Luc Duchesne^4^, Michael K. Barry^2^, Aoife J. Lowery^1,2^, Michael J. Kerin^1,2^.

**Corresponding Author:** Dr. Eoin Kerin. **Email**: [e.kerin3@universityofgalway.ie](mailto:e.kerin3@universityofgalway.ie).

**Telephone Number**: +353858143656. **ORCID ID:** 0009-0005-1627-4297.

**Address**: Discipline of Surgery, Floor 2, Lambe Institute for Translational Research, University of Galway, Galway, Ireland, H91V4AY.

**Supplementary Materials - Index**

| **Supplementary Methods** |  |
| --- | --- |
| Study Design | *page 1* |
| Eligibility Criteria | *page 2* |
| **Supplementary Figures and Tables** |  |
| Supplementary Figure 1. | *page 3* |
| Supplementary Table 1. | *page 4* |
| **References** | *page 5* |
|  |  |

**Supplementary Methods**

**Study Design**

Simon’s two-stage minimax design^1^ was employed incorporating predefined interim analysis for futility (No-Go) or continuation (Go). This model was selected to optimise ethical and resource efficiency while assessing technical performance of Wavelia following system upgrades. At α=0.05 and 80% power, 62 evaluable patients were required (30 in Stage 1, 32 in Stage 2). Success was defined as ≥44/62 lesions detected. The trial would proceed to Stage 2 if ≥18 lesions were detected in Stage 1. If fewer than 18 lesions were detected, a technical evaluation would be triggered; if justified, a new Stage 1 cohort would be enrolled. To accommodate up to 10% attrition, the enrolment cap was 103 patients. Following stage 1 and 2, data of the 62 participants were combined for analysis.

**Eligibility Criteria**

Female patients aged ≥18 years attending the Symptomatic Breast Unit with an investigator-assessed discrete breast abnormality >1 cm were eligible if they provided written informed consent, were able and willing to comply with study requirements, and could remain prone for 15–30 minutes. Participants were required to have intact breast skin, with sufficient healing if post-biopsy. A negative urine pregnancy test was required on the day of imaging for those of childbearing potential. Breast size had to permit 1–2 cm clearance within the MWBI system’s cylindrical container. Imaging was conducted ≥14 days post-biopsy for those enrolled after standard-of-care assessments.

Patients were excluded if they were pregnant, breastfeeding, had a cup size A or insufficient breast volume for MWBI, breast surgery within the previous 12 months, aesthetic breast implants, non-removable or active metallic implants (excluding biopsy clips), significant comorbidities posing risk, or were deemed unsuitable for scanning or unlikely to comply with protocol requirements.

**Supplementary Figures and Tables**


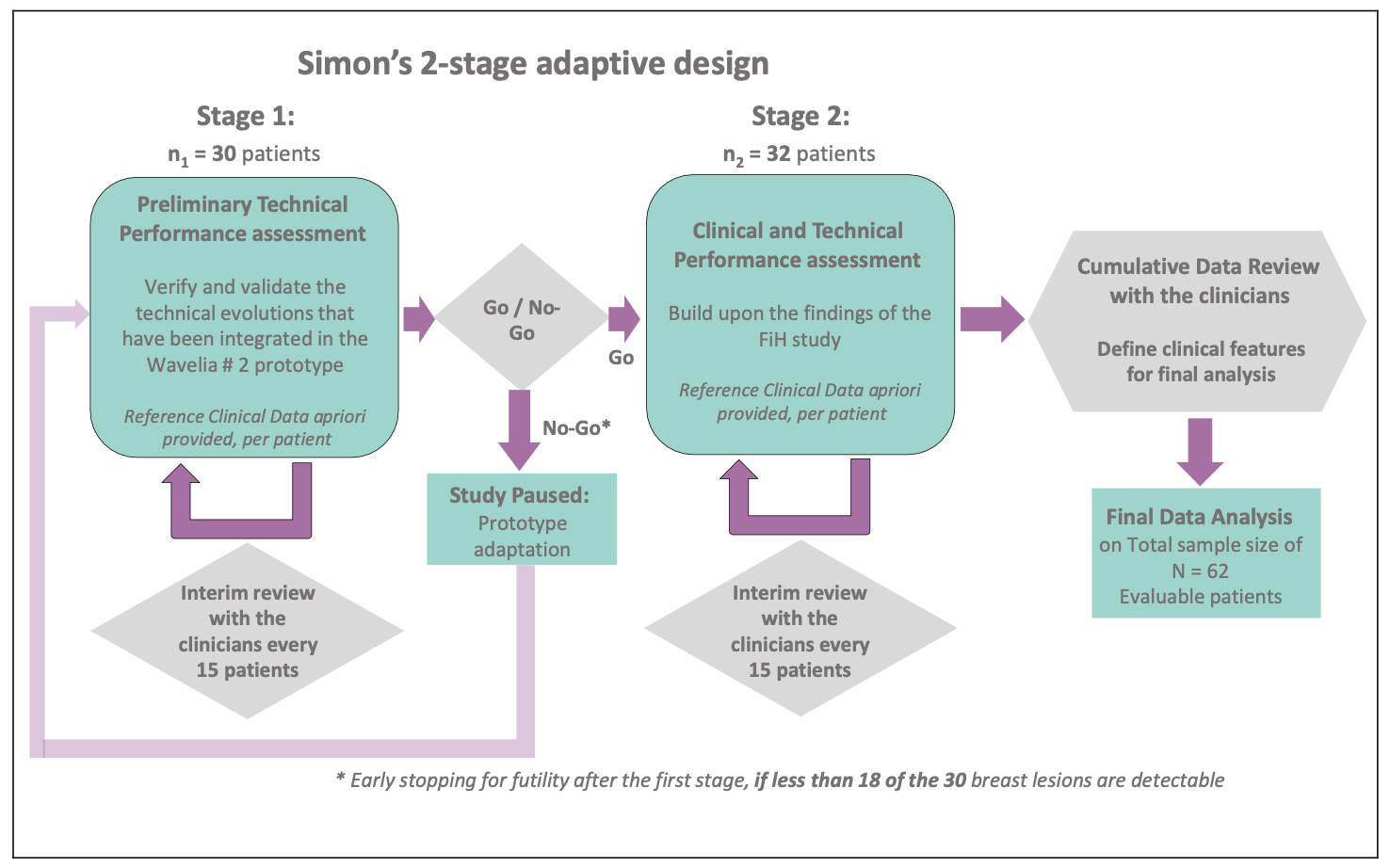


**Supplementary Figure 1.** *Schematic of the adaptive study design.*

*N; Number of patients.*

| **Patient status** | **N** |
| --- | --- |
| Screened for eligibility | 73 |
| Excluded from study | 11 |
| Integrated in Final Analysis | 62 |
| **Reason for exclusion** |  |
| - Cysts aspirated prior to Wavelia #2 scan | 1 |
| - Device deficiency (no transition liquid in scanner) | 1 |
| - Scan artefact – patient coughing during scan | 1 |
| - Scan abandoned due to discomfort in prone position | 1 |
| - Single breast scanned due to patient request (limited patient time) | 3 |
| - Breast size incompatible with scanner | 4 |

**Supplementary Table 1***. Patient Analysis Subsets, and reasons for exclusion from study.*

*N; Number of patients.*

**References**

1. Simon R. Optimal two-stage designs for phase II clinical trials. Control Clin Trials. 1989;10(1):1-10.
